# Problematic Pornography Use, Mental Health, and Suicidality among Young Adults

**DOI:** 10.3390/ijerph21091228

**Published:** 2024-09-18

**Authors:** Mujde Altin, Diego De Leo, Noemi Tribbia, Lucia Ronconi, Sabrina Cipolletta

**Affiliations:** 1 Department of General Psychology, University of Padova, 35131 Padova, Italy; mujde.altin@studenti.unipd.it; 2 De Leo Fund, 35137 Padua, Italy; d.deleo@griffith.edu.au (D.D.L.); noemi.tribbia@outlook.it (N.T.); 3 Australian Institute for Suicide Research and Prevention, Griffth University, Mt Gravatt, QLD 4122, Australia; 4 Slovene Centre for Suicide Research, Primorska University, 6000 Koper, Slovenia; 5 Computer and Statistical Services, Multifunctional Pole of Psychology, University of Padova, 35122 Padova, Italy; l.ronconi@unipd.it

**Keywords:** pornography, suicide, gender, loneliness, mental health, young adults

## Abstract

The relationship between suicidality and problematic pornography use (PPU) is not clear, and the limited research data that exists show contradictory results. The present study aims to explore the associations between these two variables in a sample of young adults, taking into account gender differences and the role of loneliness, emotional states, and well-being. A total of 332 national and international students (60% female) at an Italian University with a mean age of 23 years (SD = 2.84) completed an anonymous online survey during the period from March 2023 to May 2023. The results show that PPU was associated with higher levels of anxiety, depression, stress, loneliness, and suicide ideation, as well as lower life satisfaction. Gender comparison analysis revealed significantly higher scores for PPU and loneliness among men, while women scored higher in stress, anxiety, and life satisfaction. Significant correlations between PPU and suicidal ideation and having a suicide plan were established for female participants despite their lower scores in PPU. Multiple regression analysis revealed that PPU and its interaction with gender were both significant predictors of suicidal ideation. Future attention should be paid to how young women may be influenced by exposure to sexually explicit materials, while always considering the role of loneliness.

## 1. Introduction

Pornography consumption has dramatically risen in the last few decades [1], and the Internet has played a substantial role in this uptrend [2]. Particularly after the outbreak of the COVID-19 virus worldwide, the mandatory quarantine period caused drastic changes in several areas of individuals’ lives, including Internet pornography use [3,4]. A data report retrieved from one of the most trafficked websites globally, Pornhub, shows that with the spread of the pandemic, the traffic to Pornhub skyrocketed [5]. The number of daily new cases of COVID-19 could predict the site traffic, and the increase in Pornhub access was significantly associated with the spread of the virus [5]. Not surprisingly, this turn of events pointed to the issue of PPU, widening the interest in research on the topic [3,6,7].

The first model developed to understand better why pornography has become an appealing platform for sexual purposes was proposed by Cooper in 1998 [8]. He called this model the ‘Triple-A Engine’, in which the triple-A structure refers to the Accessibility, Affordability, and Anonymity of the Internet [8]. Thus, the fact that material can be accessed quickly and usually free of charge, while keeping the identity unsigned, may encourage its use, even in the presence of harmful effects. Potentially, those who engage with pornography to mitigate negative feelings and escape reality could be in danger of encountering problems related to its use [9].

PPU encompasses difficulties in terms of controlling the frequency, duration, or intensity of pornography use, all of which can disrupt daily functioning, relationships, or general well-being [2,10,11,12,13,14]. Some well-established negative effects related to PPU include sexual aggression [15], loneliness [13,16,17,18], distress, depression, and anxiety [2,13,19,20,21,22], neuroticism [23], sexual dissatisfaction and marital problems [24,25,26,27], negative body image [28], moral incongruence [12], low self-esteem and poor attachment [2], and isolation [29]. Moreover, in previous studies, hypersexual behaviors that usually include porn dependency showed links with executive impairment [30]. A more recent review by Castro-Calvo et al. (2021) [31] revealed that PPU shows associations more specifically with cognitive functions such as attentional bias, inhibitory control, working memory, and decision-making (e.g., a tendency to choose short-term benefits).

In the Diagnostic and Statistical Manual of Mental Disorders (DSM-5) [32] and its Text Revision (DSM-5 TR) [33], the choice of pornography content is a diagnostic indicator for some disorders but is not recognized as a separate entity [34,35]. Recent neuroscientific studies showed that there are similarities in terms of brain responses on the part of individuals with Internet addictions (including gaming, online shopping, and pornography use) and drug addictions [36,37,38,39], sparking debates about whether PPU is a form of addiction or a disorder [40]. Conversely, some feminist scholars have expressed concerns regarding the medicalization of “porn addiction” by perceiving it as an illness [41].

At this point in time, it is not very clear how we should portray and assess PPU [2,6,7,38,42,43,44]. Fernandez and Griffiths [7] investigated 22 tools assessing PPU. Among them, the Cyber Pornography Addiction Test (CYPAT) is one of those screening tools developed to provide a short and effective measure of Internet pornography addiction [7,20]. It is a brief, easily applicable, 11-item test having powerful psychometric properties. It includes 8 different addiction components: salience, impaired control, relapse, mood modification, interpersonal conflict, general life conflict, sex life conflict, and use despite harm. In addition, CYPAT is one of the two scales that evaluates the ‘use despite harm’ component as a separate category [7,20], which made it a good fit for the present study.

When discussing the effects of pornography consumption, it is often assumed that adolescents and young adults appear particularly vulnerable [45,46]. Although they may possess different motivations for using sexually explicit materials [47] due to their ongoing sexual development and curiosity, they may interpret pornographic material without critical evaluation, perceiving it as a realistic portrayal of sexuality [46,48]. For example, it was found that adolescents’ exposition to sexualized media was associated with increased sexual objectification of women [49]. Additionally, among adolescents, being male, having a sensation-seeking personality, lower self-control, lower life satisfaction, and family troubles were predictors for consuming pornography more often [10,50,51]. Efrati [52] states that the difference between having problematic or non-problematic pornography use may depend on the perspective young individuals have about mental health, feminism, or religion. This may explain consistent gender differences in pornography consumption and PPU.

In addition, previous research shows that impulsive traits commonly observed among young individuals are associated not only with PPU but also with other behavioral addictions [4,53,54]. For instance, Khatcherian et al. [55] highlighted that loneliness experienced during adolescence is linked to Internet addiction, while extreme levels of it may contribute to developing suicidal ideation. Indeed, adolescents with Internet addiction had elevated levels of depression and suicidal ideation [56]. Furthermore, in a recent review [57], researchers demonstrated the bidirectional relationship between pornography use (PU), PPU, and loneliness. PU/PPU was able to predict loneliness, and vice versa. Despite all the above-mentioned risks and concerns, intervention and treatment strategies relating to PPU among young adults are lacking, and more evidence-based research in this field is required [57].

Young individuals are also accepted to be particularly vulnerable when it comes to suicidality [58], and considering the fact that those who are at higher risk of suicide tend to spend more time on the Internet [59,60,61,62,63,64], the relationship between suicide and pornography requires more attention. A recent study of 4404 patients with behavioral addictions, including sex addiction, revealed correlations with suicidality [65]: patients with gambling disorder and sex addiction were at higher risk of developing suicidal thoughts and actively engaging in attempts. Moreover, Kim et al. [59] found that the number of adolescents who experienced depressive and suicidal episodes was highest for those who used internet pornography. In another study, suicidal men reported significantly higher use of online pornography [61]. However, it is not clear whether these participants experienced PPU specifically. These results are inconsistent with another study where no statistically significant association was found between PPU and suicidal ideation among US veteran men [66]. As highlighted by Sharpe and Mead [67], it is uncertain whether depression experienced by people who have PPU may lead to suicidality. Additionally, as mentioned earlier, the minimal data that exist on this issue show controversial results.

The aim of the present study was to explore the precise link between PPU and suicidality, but also PPU’s association with loneliness, emotional states, and life satisfaction. The focus was on young adults as a population that is particularly vulnerable to the potential negative consequences of PPU. This study included three main hypotheses:

**H1.**  *PPU is positively associated with suicidality among young adults.*

**H2.**  *PPU is positively associated with depression, anxiety, stress, and loneliness, and is negatively associated with life satisfaction.*

**H3.**  *Gender moderates the relationship between PPU and suicidality.*

We also explored the nationality/cultural background role in young adults’ PPU and mental health. Finally, loneliness was considered as a predictor that could worsen participants’ mental health if associated with PPU.

## 2. Materials and Methods

### 2.1. Participants

From March 2023 to May 2023, 337 students from the University of Padova (Italy) completed an anonymous online survey. Inclusion criteria were as follows: being 18+ years of age; being a regular or exchange student at the University of Padova; residing in Italy.

After the removal of 5 participants with missing and non-complete data, a total of 332 individuals were included in the final analysis. The mean age of the participants was 23.41 with a standard deviation of 2.84. Of these, 202 were female and 120 were male. The international sample included 64 males, 93 females, and 3 non-binary participants, whereas the Italian sample consisted of 58 males, 109 females, and 5 non-binary participants together with 2 who did not want to report their gender information. The proportion of females to males showed similarities between the total sample (about 60%), the international sample (about 58%), and the Italian sample (about 62%). Thus, the gender ratio was consistent within groups, although there was a slight imbalance in favor of female participants. The samples’ characteristics are illustrated in Table 1.

To determine the size of the necessary sample, a power analysis was conducted using G*Power; it showed that a total sample of 270 was necessary to detect a moderate effect (Slope H1 = 0.15) with a power of 0.80 to perform correlational analysis. To examine the differences between the two groups, a second power analysis was performed. The results revealed the need to recruit at least 64 participants for each group in order to conduct a T-test with a power of 0.80 and an effect size of 0.50. For both power analyses, statistical significance was set at α = 0.05.

This study was performed in line with the principles of the Declaration of Helsinki. The Ethical Committee for Psychological Research of the University of Padua approved this study (Protocol No. 5077). Those participants who gave their approval to the consent form could continue with the survey. Participants did not receive any remuneration (e.g., prize draw, course credit, etc.) for participation in this study.

### 2.2. Measures

The Qualtrics platform was used to create and distribute the survey through an online anonymous link and QR code. Both English and Italian languages were used for the survey, and participants were recruited through advertising on Facebook, Instagram, Telegram, WhatsApp, and in university campus areas. The completion of the survey took 9–10 min.

Socio-demographic information relating to the sample was gathered, and the following standardized questionnaire was administered to assess PPU, feelings of loneliness, emotional states (including depression, anxiety, and stress), and life satisfaction:

Cyber Pornography Addiction Test (CYPAT, [20]). A self-reporting scale of 11 items was scored on a five-point Likert scale, from never (0) to always (4). The summed score for the CYPAT ranges from 0 to 44, with higher scores corresponding to higher levels of PPU.

Revised UCLA Loneliness Scale (rULS-6, [68,69,70]). Initially developed for the examination of loneliness in a variety of contexts, it consists of 6 Likert scale items, ranging from never (1) to always (4). The summed score for the RULS-6 ranges from 6 to 24, and higher scores indicate higher levels of loneliness.

Depression, Anxiety, and Stress Scale (DASS-21, [71,72,73]), a short version of DASS-42 consisting of three subscales for depression, anxiety, and stress. Each subscale contains seven items, and the cut-off scores suggest the severity of the condition. All items were rated on a four-point Likert scale from 0 (did not apply to me at all) to 3 (applied to me very much or most of the time), and with the raw scores being multiplied by two. The summed score for the DASS-21 ranges from 0 to 63, with higher scores indicating higher levels of depression, anxiety, and stress.

Satisfaction With Life Scale (SWLS, [74,75]). A short 5-item scale scored on a 7-point Likert scale, ranging from completely disagree (1) to completely agree (7), designed to measure global cognitive judgments of satisfaction with one’s life. The summed score for the SWLS ranges from 7 to 35, with higher scores indicating higher levels of life satisfaction.

Four ad-hoc questions on suicidal thoughts and attempts were presented to investigate whether or not the participants had faced these experiences and, if so, how often they had occurred. For suicidal ideation, the question “Have you ever had ideas about taking your own life?” was presented (Yes/No) with four possible answers: “Never; Rarely; Sometimes; Often”. Participants who reported having suicidal ideation were additionally asked if they had a ‘specific suicide plan’ (Yes/No) with the following question: “Have you ever had a specific plan to take your own life?”. In terms of suicidal attempts, the participants were first asked, “Have you ever tried to take your own life?” and if so, how often it had happened: “Once; Twice; Two times or more”. Those who had not had any suicidal ideation in the past were not asked any further questions.

### 2.3. Data Analysis

Rstudio software was used to perform statistical analysis. The missing data were handled in various ways. Particularly, three subjects were excluded due to missing demographic information, and two more were excluded as they had missing data in all measure variables with the exception of loneliness. To handle the missing values in the CYPAT, ULS6, DASS-21, and SWLS scales, means were calculated based on the valid answers of the same subjects. The missing values were automatically excluded for group and gender comparison analysis, as well as for the correlation and regression models.

Descriptive analyses were conducted to investigate demographic variables, and Cronbach’s alpha for each questionnaire was calculated to assess the internal consistency. Gender (female vs. male) and group differences (Italian vs. international students) were investigated using Welch’s t-test for the RULS-6 and CYPAT (due to the different variance), standard t-test for DASS-21, and SWLS and Chi-square test to compare male and female scores with regard to suicidality. Spearman correlation analysis was used to measure linear relationships between variables.

Multiple regressions were computed with loneliness and PPU as independent variables and gender as a moderator to see the potential interaction effect. Suicidal ideation, depression, anxiety, stress, and life satisfaction were considered dependent variables.

## 3. Results

Descriptive statistics with regard to the questionnaire scores can be found in Table 2. Significant gender differences were found for all variables (loneliness, PPU, stress, anxiety, and life satisfaction) with the exception of depression. Female participants scored significantly higher on DASS-21 (particularly stress and anxiety) and SWLS (life satisfaction), while male participants scored significantly higher on CYPAT (problematic pornography use) and RULS-6 (loneliness). Comparisons are reported in Table 3.

Italian and international students did not report significant group differences for PPU (t = 1.196, *p* = 0.233). On the contrary, Italian students reported significantly higher levels of loneliness (t = −2.254, *p* = 0.025) and stress (t = −2.136, *p* = 0.033), while international participants scored significantly higher on life satisfaction (t = 2.055, *p* = 0.041).

Regarding the four ad-hoc questions asked to assess suicidality, 163 (49%) participants reported that they never had any suicidal thoughts, whereas the rest reported having them rarely 94 (28%), sometimes 49 (15%), and often 26 (8%). 102 (60%) did not have any specific plan to take their own life, whereas 67 (40%) specified that they did have a specific plan 32 (19%). Most participants reported that they had never attempted suicide 136 (81%), whereas 32 (19%) reported that they had attempted suicide at least once 18 (55%), twice 9 (27%), three times or more 6 (18%). No significant gender difference was found for suicidal ideation (*χ*^2^ = 2.654, *p* = 0.448), having a suicide plan (*χ*^2^ = 0, *p* = 1), or suicidal attempts (*χ*^2^ = 0.403, *p* = 0.525).

Correlations were calculated with regard to the whole sample and in the two subsamples of males and females. As reported in Table 4, PPU showed a significant relationship with all the included variables in the female sample with the exception of suicide attempts. For males, despite higher levels of reported PPU compared to females, significant associations were found with loneliness, stress, anxiety, and depression, but not in terms of life satisfaction and suicidality.

**Figure 1:**
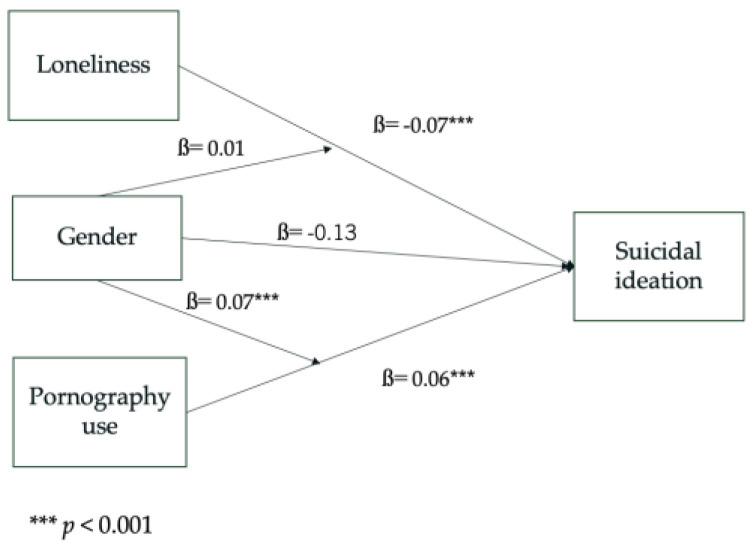
Graphic representation of the model derived by the regression analysis.

Multiple regression analysis was used to further investigate correlational findings. As shown in Table 5 and Figure 1, PPU and loneliness were used as predictors, while suicidal ideation was the dependent variable. Gender was used as a moderator to determine the potential interaction effect. Overall, the model was significant (F(5, 316) = 11.57, *p* < 0.001) and explained about 14% of the variance (considering the adjusted R2 coefficient), suggesting an optimizable fit to the data.

The individual effects of the predictors show that PPU (ß = 0.07, t = −4.44, *p* < 0.001) and loneliness (ß = 0.07, t = 3.94, *p* < 0.001) both predict suicidal ideation. Gender by itself did not significantly predict suicidal ideation (ß = −0.13, t = −0.35, *p* = 0.73), whereas its interaction with PPU showed a significant effect (ß = −0.07, t = −3.36, *p*< 0.001). Thus, the relationship between PPU and suicidal ideation is moderated by gender, and significant only for female participants. Such a moderation effect was absent with regard to loneliness (ß = 0.01, t = 0.33, *p* = 0.74).

## 4. Discussion

The aim of this study was to analyze the association between PPU and mental health, suicide ideation, and well-being in college students. Our study results showed a positive link between PPU and pornography use and anxiety, depression, stress, loneliness, and suicidal ideation, and a negative correlation with well-being. These results are in line with those of Camilleri et al. (2021) [19], who found that compulsive pornography use among university students in the US was related to mental states measured by depression, anxiety, and stress and also applied to the existing literature on loneliness [13,16,17,18].

Some important differences were found between men and women. In line with previous studies [2,25,34,42,46,76,77,78], young men reported higher scores than women in terms of cyber pornography addiction, but female participants’ PPU showed significant associations with suicidal ideation, while suicide plans were not significant in the case of male participants. Moreover, the multiple regression model results revealed that PPU and its interaction with gender (acting as a moderator) were significantly linked to suicidal ideation. Those females who had higher PPU scores were also more likely to have suicidal ideation, while for males no such effect was observed.

Due to the fact that men are accepted to be the main consumers of pornographic material, so far, the majority of the existing research has focused on male participants. Thus, it is not easy to generalize our findings or compare them with the previous literature. As mentioned previously, Shirk et al. [66] could not find any association between PPU and suicidal thoughts. Although their study focused only on male veterans, this would confirm the results of our study. Additionally, military veterans could be particularly relevant, as they experience higher levels of PPU and distress compared to non-veteran men [79].

Similarly, when mental states (stress, anxiety, depression) and life satisfaction were used as dependent variables instead of suicidal ideation, PPU was a significant predictor, and its interaction with gender was significant for stress, anxiety, and life satisfaction. In particular, those females who had higher levels of PPU showed higher stress, anxiety, and lower life satisfaction. For males, on the other hand, this was not the case. In addition, no interaction effect was found for depression, which is reasonable since no gender differences were observed for depression. Therefore, being a female and having high scores in PPU shows parallel links with psychological distress, suicidal ideation, and lower life satisfaction.

However, it is important to bear in mind that the lower occurrence of common mental health issues in men should not be directly assumed to indicate a lesser overall experience of these conditions [80]. The limited reports could be influenced by dominant ideas of hegemonic masculinity [80,81], which are based on active agency, rigidity, strength, and not showing or sharing feelings. For example, a study involving men living in rural communities in Canada revealed a paradoxical observation where higher rates of suicide and substance abuse were present alongside lower levels of depression and stress [82]. Here, stoicism may appear as an obstacle preventing men from accepting their struggles [81]. Additionally, this pattern may be different for males, who are more open to seeking help on these issues. For example, a case study of a 25-year-old single male student with an inability to stop watching porn reported that the subject experienced deteriorated relationships, poor sleep, reduced academic performance, shame, and guilt due to the PPU. This young male, who voluntarily decided to seek help and who applied to a psychiatric clinic for that purpose, reported having recurrent suicidal thoughts [83].

Another explanation for observing associations between PPU and strong negative emotions, including suicide, for females but not for males, could be the way in which the mainstream porn industry is constructed today, as its main focus is on male pleasure [84] and because of the attitudes women possess towards the sex industry and sexually explicit material in general. For instance, Shaughnessy et al. (2010) [78] showed that women report more negative attitudes towards pornography. In fact, many engaged or married women tend to condemn using porn to some extent and perceive it as being disloyal [85]. Another study shows that when exposed to the same sexual material, female participants demonstrate more negative emotional responses, suggesting notable gender differences in terms of interpretation [86]. Additionally, women have a tendency to underestimate their pornography use compared to an overestimation on the part of men, again reflecting different viewpoints [87].

Shame and guilt, as well as their interaction, experienced by women due to the consumption of pornographic material might also be a trigger for developing suicidal ideation [88]. However, it is important to highlight that there is a great deal of variation among young people on how they relate to these issues. Possibly, all interpretations should take into account the relationship to hegemonic masculinity [89].

Some other problematic and potentially addictive behaviors, such as gaming and gambling, have also been linked to suicidality among young adults [90,91,92,93]. For instance, a cross-sectional study conducted on young adults in Great Britain showed that gambling is linked to suicide attempts for both genders [93]. Similarly, gaming has been linked to suicidal ideation, mediated by emotion and moderated by hope [90]. These studies, together with our results, highlight the fact that the behavioral patterns associated with addictive components are extremely risky, especially for younger age categories, and may have serious consequences, including suicidality.

The role of loneliness was also explored in the present study. In addition to correlational findings, the results of the multiple regression analysis showed that loneliness and PPU were both significant predictors of suicidal ideation. The relationship between loneliness and suicidality is a well-established one in the literature [55,94,95,96], and our findings are in line with them. As previously reported by Batigun (2005) [97], loneliness is an age-dependent factor and is common among young adults. Interestingly, the interaction effect with gender (acting as a moderator) on suicidal ideation was observed only with regard to PPU, and not in the case of loneliness, despite the significant gender differences (males scored significantly higher than females). Likewise, loneliness was a significant predictor of mental states (stress, anxiety, depression) and life satisfaction, but an aspect that does not show any interaction with gender. Experiencing elevated levels of loneliness therefore appears as a critical factor that should be considered in the context of mental health, regardless of gender.

Ahorsu et al. [98], during their review on problematic porn use and cross-cultural differences, pointed out that European, American, and Oceanian countries have more up-to-date and in-depth literature on pornography, more liberal beliefs and attitudes, as well as specialized treatment protocols and facilities. Asian countries are similar producers of the literature on this topic; however, they are conservative in their beliefs and attitudes toward pornography, which has limited options and specialized treatment facilities for people with PPU. African countries share similarities with Asian countries except for the paucity of the literature on pornography. In our study instead, regarding nationality/cultural differences, no significant results were obtained, either for suicidality or for PPU in a comparison of Italian and international students. However, the former had higher loneliness and stress levels, together with lower levels of life satisfaction. Previous studies have found that international students run a higher risk of developing mental issues compared to domestic ones [99,100,101], which seems to contradict our results. However, it is important to note that our sample included both long-term and short-term international students and did not explore the potential differences between them. The literature shows that long-term international students compared to short-term international students (exchange students participating in Erasmus+, ULISSE programs, etc.) face more psychological problems and difficulties [102,103]. This may explain why, in our sample, Italian domestic students had a worse psychological profile in general.

### Limitations

This study suffers from several limitations. First, it is important to highlight that our study had a cross-sectional design, which makes it impossible to infer any causal relation between variables.

Second, the PPU assessment tool used in this study, CYPAT, as well as other measurement tools developed in recent years, usually investigate different components of PPU, such as frequency and intensity, but not the consumed content of pornography, such as violent, nonviolent, or degrading. This may appear as a limitation of this study since the various types of content are associated with different factors (e.g., sexual aggression) [15].

In addition, the present study highlights important gender differences with regard to the investigated variables while lacking information about sexual orientation. As highlighted by Borgogna et al. (2022) [104], sexual minorities report higher levels of PPU compared to heterosexual males and females. Thus, future studies should consider gender and sexual orientation together.

Due to the sensitive nature of the research topic, an anonymous online survey was chosen as a data collection method. All participants were asked to sign a consent form in which they were informed about the anonymity, confidentiality, and security of their data. However, due to the self-report design, it is possible that collected data include some biases, which potentially could distort the results. It is also possible that some participants may have provided non-complete information as a result of the delicate nature of the topics under consideration. For these reasons, the results should be interpreted carefully.

Another important point to bear in mind is that since the legal use and accessibility of pornography will vary across countries, cross-cultural studies should replicate our findings by comparing the different environments in which pornography is manufactured and consumed. Future research may also include detailed qualitative research to obtain a more comprehensive view of PPU and suicide relationships on the part of young individuals.

Additionally, using a longitudinal design for future studies may be beneficial to follow how the temporal relationship between PPU and mental health outcomes changes over the years. Finally, it is crucial to consider factors such as religion or moral values when studying PPU, as they may affect the individual’s approach to pornography in general [6,105,106].

## 5. Conclusions

To the best of our knowledge, the present study is the first one focusing explicitly on the relationship between PPU and suicidality. Our sample was composed of young adults studying in Italy. Despite lower levels of reported PPU compared to males, a significant relationship was found for female participants only. In addition, the PPU scores of females were linked to loneliness, stress, anxiety, depression, and life satisfaction. For males, on the other hand, PPU scores were linked to loneliness, stress, anxiety, and depression. Therefore, it can be stated that females are at particular risk of developing suicidal ideation due to PPU. Moreover, loneliness plays a crucial role as a predictor of suicidality for both genders. Future research should pay more attention to how young women may be influenced by exposure to sexually explicit materials, always taking into account the role of loneliness. Another idea could be to investigate more ‘female-friendly’ sexual materials, such as audio erotica to check if the results are consistent. Finally, the findings of our study should be replicated for older women, as they are the least studied category in the given context.

These findings, after being further explored by future studies, could have a significant impact on structuring new mental health protocols and subsequent clinical practice. For example, in public health policies, support pathways for addiction and/or suicidality could be created by taking gender differences into account. As suggested by Antons et al. [107], incorporating them into protocols structured around them and based on CBT (cognitive behavior therapy) could lead to more specific and effective programs to support people experiencing these issues. The topics discussed in this article represent taboos in our society. Regarding prevention, one could therefore try to solicit people’s awareness to the population both about the association between PPU and suicidal thoughts (allowing it to be identified as a warning signal for seeking psychological support) and about gender differences in pornography consumption and the cultural context in which these issues are immersed. In clinical practice, one option might be to work on fostering awareness and emotional expression. One way might be using self-help groups [108] to share experiences, reduce the sense of loneliness and isolation, and promote sociality.

## Figures and Tables

**Table 1:** Samples’ Characteristics.

Factor Respondents	N (%)
Gender	
Female	202 (61%)
Male	120 (36%)
Non-binary	8 (2%)
Prefer not to say	2 (1%)
Group	
Italian	178 (54%)
International	154 (46%)
Field of Study	
School of Agricultural Sciences and Veterinary Medicine	13 (4%)
School of Economics and Political Science	15 (5%)
Law School	12 (4%)
School of Engineering	37 (11%)
School of Medicine	15 (5%)
School of Psychology	108 (33%)
School of Science	66 (20%)
School of Human and Social Sciences and Cultural Heritage	28 (8%)
Others	39 (12%)

**Table 2:** Descriptive Statistics of Questionnaires’ Scores.

	Cronbach’s Alpha	Mean	Std. Deviation	Skewness	Kurtosis
CYPAT	0.85	3.94	5.18	2.097	5.421
rULS-6	0.83	13.45	3.76	0.228	−0.538
DASS-21	0.86	15.04	9.51	0.488	−0.431
SWLS	0.86	21.75	6.46	−0.289	−0.571

**Table 3:** Gender differences in the present study variables.

Scale	Gender	Mean	t-Value	df	*p*-Value Cohen’s d
CYPAT (PPU)	Female	2.50	−6.317	191.32	<0.001 −0.91
	Male	6.37			
rULS-6 (loneliness)	Female	15.37	−5.936	196.67	<0.001 −0.85
	Male	20.65			
DASS-21 (stress)	Female	16.35	3.556	320	<0.001 0.40
	Male	12.52			
DASS-21 (anxiety)	Female	10.13	3.005	320	0.003 0.34
	Male	7.15			
DASS-21 (depression)	Female	12.77	−0.186	320	0.852 −0.02
	Male	12.99			
SWLS (life satisfaction)	Female	22.67	3.136	320	0.002 0.35
	Male	20.37			

**Table 4:** Correlations between the main variables in the total sample and by gender.

	1	2	3	4	5	6	7	8	9
1. CYPAT	-	0.22 ***	0.11 *	0.14 *	0.23 ***	−0.18 **	0.19 ***	0.08	0.06
Males	-	0.33 ***	0.23 *	0.19 *	0.28 **	−0.04	0.11	−0.06	−0.04
Females	-	0.14 *	0.26 ***	0.30 ***	0.28 ***	−0.17 *	0.32 ***	0.19 **	0.10
2. rULS-6	-	-	0.41***	0.38 ***	0.57 ***	−0.48 ***	0.26 ***	0.20 ***	0.11 *
Males	-	-	0.50 ***	0.37 ***	0.60 ***	−0.42 ***	0.36 ***	0.12	0.14
Females	-	-	0.39 ***	0.42 ***	0.55 ***	−0.51 ***	0.24 ***	0.26 ***	0.14 *
3. DASS-21 (stress)	-	-	-	0.65 ***	0.72 ***	−0.41 ***	0.32 ***	0.27 ***	0.14 *
Males	-	-	-	0.60 ***	0.77 ***	−0.50 ***	0.33 ***	0.25 **	0.17
Females	-	-	-	0.65 ***	0.70 ***	−0.43 ***	0.30 ***	0.26 ***	0.15 *
4. DASS-21 (anxiety)	-	-	-	-	0.60 ***	−0.31 ***	0.30 ***	0.26 ***	0.17 **
Males	-	-	-	-	0.56 ***	−0.30 ***	0.21 *	0.20 *	0.20 *
Females	-	-	-	-	0.63 ***	−0.36 ***	0.33 ***	0.30 ***	0.19 **
5. DASS-21 (depression)	-	-	-	-	-	−0.57 ***	0.38 ***	0.24 ***	0.14 *
Males	-	-	-	-	-	−0.62 ***	0.37 ***	0.17	0.11
Females	-	-	-	-	-	−0.55 ***	0.37 ***	0.29 ***	0.19 **
6. SWLS	-	-	-	-	-	-	−0.31 ***	−0.25 ***	−0.13 *
Males	-	-	-	-	-	-	−0.15	−0.11	−0.04
Females	-	-	-	-	-	-	−0.42 ***	-0.34 ***	−0.19 **
7. Suicidal Ideation	-	-	-	-	-	-	-	0.58 ***	0.45 ***
Males	-	-	-	-	-	-	-	0.55 ***	0.44 ***
Females	-	-	-	-	-	-	-	0.57 ***	0.43 ***
8. Suicide Plan	-	-	-	-	-	-	-	-	0.60 ***
Males	-	-	-	-	-	-	-	-	0.58 ***
Females	-	-	-	-	-	-	-	-	-
9. Suicide Attempt	-	-	-	-	-	-	-	-	-
Males	-	-	-	-	-	-	-	-	-
Females	-	-	-	-	-	-	-	-	-

* *p* < 0.05, ** *p* < 0.01, *** *p* < 0.001. Note. CYPAT (Cyber Pornography Addiction Test); rULS-6 (6-item Revised UCLA Loneliness Scale); DASS-21 (Depression Anxiety Stress Scales Short Version); SWLS (Satisfaction With Life Scale).

**Table 5:** Multiple linear regression model with interaction.

Coefficients	Estimate	Standard Error	t-Value	*p*-Value
Intercept	−0.24	0.23	−1.02	0.31
CYPAT	0.07	0.01	4.44	<0.001
rULS-6	0.07	0.08	3.94	<0.001
Gender (moderator)	−0.13	0.37	−0.35	0.72
CYPAT * Gender	−0.07	0.02	−3.36	<0.001
RULS-6 * Gender	0.01	0.03	0.33	0.74
R^2^	0.15			
F(5, 316)	11.57			<0.001

Note. Dependent variable: suicidal ideation.

## Data Availability

The data that support the findings of this study are openly available in OSF at https://osf.io/9udz5/ (accessed on 30 July 2024).
